# *Candida/Staphylococcal* Polymicrobial Intra-Abdominal Infection: Pathogenesis and Perspectives for a Novel Form of Trained Innate Immunity

**DOI:** 10.3390/jof5020037

**Published:** 2019-05-09

**Authors:** Shannon K. Esher, Paul L. Fidel, Mairi C. Noverr

**Affiliations:** 1 Center of Excellence in Oral and Craniofacial Biology, School of Dentistry, Louisiana State University Health Sciences Center, New Orleans, LA 70119, USA; sesher1@tulane.edu (S.K.E.); pfidel@lsuhsc.edu (P.L.F.J.); 2 Department of Microbiology and Immunology, School of Medicine, Tulane University, New Orleans, LA 70112, USA

**Keywords:** Sepsis, polymicrobial, *Candida*, *Staphylococcus*, trained innate immunity, MDSC

## Abstract

Polymicrobial sepsis is difficult to diagnose and treat and causes significant morbidity and mortality, especially when fungi are involved. In vitro, synergism between *Candida albicans* and various bacterial species has been described for many years. Our laboratory has developed a murine model of polymicrobial intra-abdominal infection with *Candida albicans* and *Staphylococcus aureus*, demonstrating that polymicrobial infections cause high levels of mortality, while monoinfections do not. By contrast, closely related *Candida dubliniensis* does not cause synergistic lethality and rather provides protection against lethal polymicrobial infection. This protection is thought to be driven by a novel form of trained innate immunity mediated by myeloid-derived suppressor cells (MDSCs), which we are proposing to call “trained tolerogenic immunity”. MDSC accumulation has been described in patients with sepsis, as well as in in vivo sepsis models. However, clinically, MDSCs are considered detrimental in sepsis, while their role in in vivo models differs depending on the sepsis model and timing. In this review, we will discuss the role of MDSCs in sepsis and infection and summarize our perspectives on their development and function in the spectrum of trained innate immune protection against fungal-bacterial sepsis.

## 1. Introduction

Invasive fungal infections are estimated to cause over 1.5 million deaths per year [1]. Among these, *Candida* species are the most common cause of invasive fungal infections worldwide, with invasive candidiasis manifesting as multiple diseases ranging from disseminated candidiasis and candidemia to intra-abdominal candidiasis. Intra-abdominal infections (IAI) with *Candida* originate from the outgrowth and entry of organisms from the GI tract into the abdominal cavity. These infections can result in a variety of clinical manifestations, from localized peritonitis to disseminated infection, leading to lethal sepsis [2]. IAIs are often polymicrobial [3,4] and those involving fungi are associated with worse outcomes, increased antimicrobial use, and higher mortality compared to mono- or polymicrobial bacterial only infections [5,6,7,8,9,10]. Fungal involvement also leads to increased rates of relapse and more severe disease scores [8,9,11]. Despite this, the clinical significance of *Candida* isolation from the abdominal cavity is debated and likely depends on many factors, including the source (community-acquired versus nosocomial-acquired) and type of IAI (e.g., intra-abdominal abscess, peritonitis, gastrointestinal perforation) [5,12,13]. As a result, while preemptive antifungal therapy has been shown to improve survival in bacterial IAI patients [13], *Candida* is only treated as a causative infectious agent in most patients if they are immune compromised or have had prolonged exposure to antibiotics. 

### 1.1. Synergism between Candida and Bacteria

**Figure 1:**
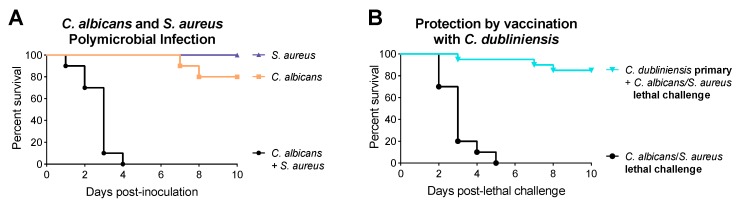
Representative graphs of (**A**) *Candida albicans/Staphylococcus aureus* synergistic lethality and (**B**) *Candida dubliniensis*-induced protection. Adapted from [20,21,23].

Synergistic effects have been reported between *Candida* and various bacteria, including both gram-positive and gram-negative organisms. As early as 1958, Yamabayashi et al. reported that mixed inoculations of *Candida albicans* with *Proteus vulgaris* or *Pseudomonas aeruginosa* caused increased mortality in mice [14]. Similar synergism has been reported for *Mycobacterium tuberculosis* [15], as well as enteric pathogens including *Staphylococcus aureus* [16], *Serratia marcescens* and *Streptococcus faecalis* [17], *Escherichia coli* [18], and *E. coli/Bacteroides fragilis* [19]. Using an animal model of polymicrobial IAI developed in our lab several years ago, we have also shown synergy between *C. albicans* and *S. aureus*. While monoinfection with either organism is not fatal, coinfections with *C. albicans* and *S. aureus* lead to 100% mortality by 48 h (Figure 1A) [20,21]. Mortality is associated with a significant increase in local and systemic proinflammatory cytokines, but not with increased microbial burden or *Candida* morphogenesis [20,21,22]. Further studies demonstrated that this synergistic lethality was not unique to *C. albicans* and also occurred with various non-albicans *Candida* species (NAC), including *Candida krusei* and *Candida tropicalis*. On the other hand, coinfections with *Candida dubliniensis*, *Candida glabrata*, *Candida parapsilosis*, and *Saccharomyces cerevisiae* resulted in minimal mortality [21,23]. Overall, we found that synergism amongst NAC species was not associated with the ability to form true hyphae, as *C. krusei* (no hyphae) was synergistically lethal during coinfection with *S. aureus*, while *C. dubliniensis*, a close phylogenetic relative of *C. albicans* that forms hyphae in vivo, was not synergistically lethal. 

### 1.2. C. dubliniensis-Mediated Protection against Polymicrobial Sepsis

The fact that closely related *C. dubliniensis* was not synergistically lethal during polymicrobial IAI prompted investigation of its potential for inducing protective immunity. Interestingly, we found that the rechallenging of *C. dubliniensis*/*S. aureus*- or *C. dubliniensis*-infected mice with lethal *C. albicans/S. aureus* 14 days later led to 80−90% protection (Figure 1B) and that this protection was long-term (up to 60 days between primary *C. dubliniensis* challenge and lethal *C. albicans*/*S. aureus* challenge) [23]. However, mice deficient in T and B cells (RAG1^−/−^ mice) maintained this protection, indicating that it was not mediated by adaptive immunity [23]. This suggested a role for trained innate immunity (TII), which refers to a non-specific memory immunity mediated by innate cells that have been “trained” by an initial challenge, leading to an enhanced response to a secondary challenge [24,25]. TII has typically been described in the context of trained monocytes/macrophages, however, we found that mice depleted of macrophages prior to the lethal rechallenge were also protected [23], indicating that the TII response induced in our model was mediated by a different innate cell type. In previous work, we observed a significant influx of polymorphonuclear (PMN) leukocytes, specifically neutrophils, by hematoxylin and eosin (H&E) staining in the peritoneal cavity following lethal *C. albicans*/*S. aureus* infection [20]. We confirmed and quantified this influx by flow cytometry using the mouse granulocyte differentiation antigen-1 (Gr-1), which is commonly used to identify neutrophils, but binds to both Ly6G (expressed by neutrophils) and Ly6C (expressed by neutrophils, dendritic cells, and monocytes/macrophages) [26]. In studies to investigate whether a similar recruitment occurred in mice that had received a *C. dubliniensis* primary challenge, we observed a similar increase in PMNs in the peritoneal cavity, as well as increased levels of Gr-1^+^ F4/80^−^ (mouse macrophage marker) leukocytes in the spleens and bone marrow of mice given a *C. dubliniensis* primary challenge, compared to naïve mice prior to *C. albicans/S. aureus* challenge [23]. We found that protection was dependent on these Gr-1^+^ leukocytes, as survival was significantly abrogated in mice treated with anti-Gr-1 depleting antibodies [23]. Because neutrophils are very short-lived cells, we considered that another Gr-1^+^ PMN cell type may be providing the long-term protection observed in our model. Myeloid-derived suppressor cells (MDSCs) can be phenotypically similar to PMNs/neutrophils, express Gr-1^+^, and, as discussed below, have been reported in sepsis models. MDSCs are a phenotypically heterogeneous (granulocytic/Ly6G^+^ or monocytic/Ly6C^+^) population of Gr-1^+^ CD11b^+^ immature myeloid cells with anti-inflammatory functions, including, most notably, T cell suppression. In unpublished data, we have demonstrated that the protective Gr-1^+^ cell population exhibits MDSC-like phenotypes. In particular, using T cell proliferation assays, we have shown that Gr-1^+^ cells isolated from protected mice have T cell suppressive activity (E.A. Lilly, unpublished data). We therefore propose that *C. dubliniensis* induces a novel form of TII mediated by MDSCs to protect against lethal polymicrobial IAI. In this review, we will consider the characteristics, types, and development of MDSCs, as well as their roles in sepsis and infection and, finally, provide perspectives on their potential role and mode of action against IAI/sepsis. 

## 2. Myeloid-Derived Suppressor Cells

The term myeloid-derived suppressor cell, or MDSC, was proposed by Gabrilovich et al. to describe an undefined population of immunosuppressive myeloid cells recently identified in association with various pathologic conditions, including infection, sepsis, inflammation, traumatic stress, and, most prominently, cancer [27]. Several excellent reviews have covered detailed aspects of MDSC differentiation and function [28,29,30,31]. On a very basic level, MDSCs are a heterogeneous population of myeloid cells with suppressive functions. These cells share several common characteristics, including the expression of Gr-1 and CD11b in mice coupled with the lack of expression of maturation markers, the inability to differentiate into mature myeloid cells, high levels of reactive oxygen species (ROS) and arginase 1 expression, and the ability to suppress immune responses both in vitro and in vivo [27]. 

Before the term MDSC was proposed, these immunosuppressive myeloid cells were referred to by several other names in the literature, including natural suppressor cells, immature myeloid cells, and suppressor macrophages [32]. MDSCs arise and develop following the normal myelopoietic pathway and are induced by similar normal growth factors. However, because they are activated in a way that is distinct from normal myeloid activation, MDSCs do not result simply from the expansion of immature myeloid progenitors (Rev. in [30,33,34]). Compared to their mature myeloid counterparts, such as neutrophils and monocytes, MDSCs are much less phagocytic and produce high levels of ROS, nitric oxide (NO), and anti-inflammatory cytokines, in addition to being immunosuppressive [30,33]. 

### 2.1. MDSC Subsets

MDSCs are composed of two subtypes, granulocytic or polymorphonuclear MDSCs (G/PMN-MDSCs) and monocytic MDSCs (M-MDSCs) [29,35,36]. In mice, G-MDSCs are CD11b^+^ Ly6G^+^ Ly6C^low^, whereas M-MDSCs are CD11b^+^ Ly6G^−^ Ly6C^high^. M-MDSCs have also been shown to express higher levels of F4/80, CD115, and CCR2 [36]. While both subsets suppress antigen-specific T cell responses, they do so through different mechanisms. G- and M-MDSCs express comparable amounts of arginase 1, while G-MDSCs produce higher levels of ROS and M-MDSCs preferentially produce NO [35,36]. G-MDSCs or M-MDSCs can be preferentially expanded depending on the stimulus/model/disease, however, M-MDSCs have been shown to be more immunosuppressive. 

### 2.2. Development of MDSCs

Healthy murine bone marrow contains around 20–30% CD11b^+^ Gr-1^+^ cells. These cells are rapidly and efficiently differentiated into mature cells, maintaining steady state cellular levels. By contrast, CD11b^+^ Gr-1^+^ cells have been shown to represent up to 90% of the cells in the bone marrow during sepsis [37]. The development of MDSCs has been proposed to occur in two steps: expansion and activation [38]. During the expansion step, the population of immature myeloid cells within the bone marrow is expanded, in part due to a block in further differentiation of these cells. The signals inducing expansion are primarily factors produced by tumor cells and include growth factors and cytokines, such as granulocyte-macrophage colony-stimulating factor (GM-CSF) [39,40], granulocyte colony-stimulating factor (G-CSF) [41], macrophage colony-stimulating factor (M-CSF) [42], IL-6 [43], vascular endothelial growth factor (VEGF) [44], stem cell factor (SCF) [45], and prostaglandins [46,47]. The expansion of MDSCs has been shown to be mediated primarily through STAT3 [48,49]. STAT3 activation also leads to the expression of S100A8 and S100A9, which contribute to blocking the differentiation of immature myeloid cells, further enhancing MDSC expansion [50,51]. During the activation step, this expanded immature population becomes pathologically activated and their suppressive functions are expressed. MDSC activation is induced by factors produced by activated T cells and tumor stromal cells, as well as proinflammatory cytokines such as IFNγ [35,52], IL-4 [53], IL-13 [52,54], TGF-β, and various toll-like receptor (TLR) ligands [37,55,56,57,58]. MDSC activation is mediated by multiple signaling pathways, including STAT6, STAT1, and primarily NFκB signaling [30,38]. A role for TLR signaling through MyD88 in activating MDSCs has also been described [37]. It is unclear whether MDSCs are activated within the bone marrow and then travel to tumor or inflammatory sites, or if the immature cells are recruited and activated at extramedullary sites. Nevertheless, mature MDSCs have been isolated from the blood, spleen, liver, lung, and tumors of mice, and the blood, tumors, and bone marrow of humans. 

### 2.3. Mechanisms of MDSC Immunosuppression

MDSCs have the capacity to suppress many types of immune cells, but they most commonly act on T cells. Suppression generally occurs through direct contact between T cells and MDSCs, but it can also occur through the combination of a variety of mediators. As mentioned previously, the most well described effectors expressed by MDSCs are arginase 1, inducible nitric oxide synthase (iNOS), and ROS. The suppressive activities of arginase and iNOS are associated with L-arginine metabolism, which is a substrate for both arginase 1 and iNOS and required for proper T cell function [59,60]. Depletion of L-arginine affects T cells in multiple ways, including by disrupting T cell receptor mediated signaling and the cell cycle [59]. The utilization of L-arginine by iNOS also results in the production of NO, which, in addition to suppressing T cell function through various mechanisms, can combine with ROS to produce peroxynitrate, which can inhibit downstream signaling through the nitration of T cell receptors and CD8 molecules, further impairing T cell binding and function [61,62]. M-MDSCs have been shown to express higher levels of arginase 1 and NO, while G-MDSCs preferentially express ROS and arginase 1 [35,36]. MDSCs also produce increased levels of IL-10 and TGF-β and promote the expansion of regulatory T cells (Rev. in [63]). 

### 2.4. Limitations of Studying MDSCs

Several limitations exist in terms of studying MDSCs. The first is that MDSCs closely resemble other types of innate cells and specific markers to discriminate MDSCs are not currently well characterized. For these reasons, phenotype and/or morphology alone are not sufficient to identify MDSCs. To date, the gold standard for definitive identification of MDSCs is by demonstrating their immunosuppressive function through T cell proliferation assays. Still, many studies do not functionally characterize their cells of interest, making interpretation difficult. Furthermore, the nomenclature of cells with MDSC-like phenotypes prior to the introduction of the term MDSC has added to the confusion [32]. 

Studying MDSCs in human populations presents even more difficulties, as cells cannot be isolated from lymphoid organs, but rather have to be isolated from peripheral blood. This makes studying MDSC expansion and site of activation in humans nearly impossible. Additionally, humans do not express Gr-1, making phenotypic analysis more complicated as well. Like murine MDSCs, there are granulocytic and monocytic subsets of human MDSCs. While a large number of markers have been identified [64], in general human G-MDSCs are CD11b^+^ CD14^−^ CD15^+^ or CD11b^+^ CD14^−^ CD66b^+^ and M-MDSCs are CD11b^+^ CD14^+^ HLA-DR^−/lo^ CD15^−^ [40,65]. A third subset of “early stage” or eMDSCs that are Lin^−^ HLA-DR^−^ CD33^+^ and composed of more immature progenitor cells has also been described in humans [66].

## 3. Role of MDSCs in Sepsis and Infection

While much of the initial work on MDSCs was carried out in relation to cancer, more recent data has demonstrated that these cells are also present and relevant in infections and sepsis (Rev. in [63,67,68]). MDSCs have been shown to accumulate in a number of bacterial infections, including *S. aureus* [69,70,71], *Mycobacterium tuberculosis* [72,73,74,75,76], and *Pseudomonas aeruginosa* [57]. However, whether the accumulation of these cells is beneficial or harmful is unclear and depends on the bacteria. MDSC accumulation has also been reported in fungal infections, including *Aspergillus fumigatus* and *C. albicans* [77]. Clinically, MDSCs are generally considered to be detrimental to the host [68]. Studies have demonstrated that high levels of MDSCs in patients are associated with an increased risk of nosocomial infection [78], longer intensive care unit (ICU) stays, worse outcomes, and earlier mortality [79]. In particular, Uhel and colleagues found that G-MDSCs were specifically increased in sepsis patients, compared to other ICU patients, and that high levels of G-MDSCs and arginase 1 early after the onset of infection were predictors for subsequent nosocomial infections [78]. 

Delano and colleagues were the first to identify MDSCs in a sepsis model, showing increased Gr-1^+^ CD11b^+^ cell populations in the spleen, lymph nodes, and bone marrow during polymicrobial sepsis [37]. They further demonstrated that this was MyD88-dependent and that MDSC accumulation was associated with suppressed T cell function and Th2 polarization. Using an anti-Gr-1 antibody, they showed that MDSC depletion prevented Th2 skewing and reversed suppressed T cell functions [37]. Several more recent studies have demonstrated that MDSC accumulation in sepsis is beneficial to the host. Noel et al. demonstrated that when MDSCs were depleted by gemcitabine treatment, mice with experimental burns lost their resistance to secondary *P. aeruginosa* infection [80]. Hepatocyte-specific deletion of the IL-6 family receptor, gp-130, abolished MDSC accumulation and mobilization and resulted in increased mortality in a mouse model of polymicrobial sepsis [81]. Furthermore, adoptive transfer of MDSCs to gp130-deficient mice provided protection against sepsis-associated mortality [81]. 

Sepsis occurs in two phases, the first of which is characterized by an initial acute hyperinflammatory phase, followed by a secondary hypoinflammatory and immunosuppressive phase. As such, several sepsis models have demonstrated that MDSCs are beneficial in sepsis in a time-dependent manner, depending on the stage of sepsis. Derive et al. demonstrated that MDSCs isolated from early and late stage sepsis have different functions. They found that, compared to MDSCs isolated three days after the onset of sepsis, MDSCs isolated 10 days after sepsis were highly functional, with robust cytokine and ROS production and arginase 1 activity. This was despite the fact that both MDSC populations could inhibit T cell proliferation in vitro. In agreement with these observations, adoptive transfer of day 10 MDSCs, but not day three MDSCs, was protective against a polymicrobial sepsis challenge [82]. Similarly, Brudecki et al. found that adoptive transfer of early MDSCs increased the proinflammatory response and resulted in greater early sepsis mortality, while transfer of late MDSCs induced anti-inflammatory cytokine production during early sepsis [83]. They also found that early and late MDSCs expressed different effectors; early MDSCs produced more NO and proinflammatory TNFα and IL-6, while late MDSCs had increased arginase 1 activity and produced anti-inflammatory IL-10 and TGF-β. Interestingly, they also observed that the late MDSC population had a greater percentage of cells that were positive for CD31, a marker for early myeloid cells. These cells also lacked the ability to differentiate further when stimulated with GM-CSF, suggesting that this late MDSC population is more immature and immunosuppressive than the early MDSCs [83]. 

MDSC accumulation has also been demonstrated in association with fungal infections. Rieber et al. found that MDSCs could be isolated from patients with *C. albicans* and *A. fumigatus* infections [77]. They further demonstrated that *C. albicans* and *A. fumigatus* could induce the differentiation of functional MDSCs from human peripheral blood mononuclear cells (PBMCs) and murine bone marrow cells, and that MDSCs could be isolated from various organs of mice infected with either pathogen. They determined that MDSC induction was dependent on the dectin-1/Syk/Card9 pathway, as well as downstream factors including ROS, caspase-8 activity, and IL-1β production. Additionally, adoptive transfer of MDSCs was able to protect against *C. albicans*, but not *A. fumigatus* infection. In follow up studies, Singh et al. demonstrated that MDSCs could be induced from human PBMCs differentially by other NAC species, including *C. glabrata, C. krusei*, and *C. dubliniensis* [84]. Although MDSCs have been demonstrated to be induced clinically and in experimental models of fungal infection, the role for these cells in protection versus pathology of infection is not entirely clear. However, we have demonstrated that fungal-induced putative MDSCs exert a protective role against lethal sepsis in our model of polymicrobial IAI [23], which warrants further exploration.

## 4. Recent Advances in MDSC-Mediated Trained Innate Immunity against Polymicrobial IAI

### 4.1. Properties of C. dubliniensis-Mediated Trained Innate Immune Protection

To further explore *C. dubliniensis*-induced trained innate protection mediated by MDSCs, we sought to define the properties and requirements of this protection. In addition to our previous observations that *C. parapsilosis* and *C. glabrata* could provide similar levels of protection [21], we have now shown that this protection also extends to *C. auris, S. cerevisiae*, and the yeast-locked *C. albicans efg1*∆/∆ *cph1*∆/∆ strain [85]. All of these strains are considered to be low virulence in our polymicrobial IAI model, which is in contrast to wild type *C. albicans*, *C. tropicalis*, and *C. krusei*, which are highly lethal in our coinfection model [21] and do not provide appreciable levels of protection [23]. We have also now demonstrated that the protection can be induced by *C. dubliniensis* as early as seven days prior to lethal coinfection and protection can be maintained through multiple lethal rechallenges up to 20 days apart [85]. We have also observed that the standard *C. dubliniensis* intraperitoneal primary challenge can protect against a lethal *C. albicans* intravenous bloodstream infection, but not against a *C. albicans* mucosal vaginal infection [85]. 

### 4.2. Pathogen Manipulation of the Hematopoietic Compartment

Of particular interest, we have recently been able to culture *Candida* from the bone marrow of intraperitoneally inoculated mice [85]. *C. dubliniensis*-inoculated animals had the highest fungal infiltration after 24 hours; however, we were also able to detect *C. albicans*, other NAC species including *C. auris* and *C. glabrata*, and *S. cerevisiae* in the bone marrow of mice. After 48 hours, the level of fungal persistence was positively correlated with the average level of protection reported in our polymicrobial IAI model. These data suggest that the ability of these species to access and persist in the bone marrow may be related to their protective potential. This is an intriguing finding that fits into a bigger picture that has recently emerged on how pathogens may manipulate the hematopoietic compartment and ultimately impact the innate immune response. Several pathogen infections have been shown to influence the hematopoietic stem and progenitor cell (HSPC) population, including *Candida* [86,87,88], *E. coli* [89,90,91], *P. aeruginosa* [92], *Ehrlichia chaffeensis* [93], *Anaplasma phagocytophilum* [94], *Listeria monocytogenes* [95], *Mycobacterium* [96,97], and several viral infections . Furthermore, the HSPC population has been shown to be expanded in a model of polymicrobial sepsis [98]. 

While changes to HSPCs in the setting of infection have traditionally been considered a reactive process, more recent work has demonstrated that these cells may directly interact with and respond to pathogens. In support of this concept and in agreement with our findings, Yanez et al. demonstrated that after intravenous inoculation, *C. albicans* could access the bone marrow and directly stimulate HSPCs through TLR2 and possibly dectin-1 [88]. In more recent work, Kaufmann et al. demonstrated that the bacillus Calmette-Guérin (BCG) vaccine strain could access the bone marrow and manipulate the transcriptional signature of HSPCs [97]. This resulted in an expanded macrophage population that was found to have epigenetic modifications that rendered them more effective at killing virulent *M. tuberculosis* compared to naïve macrophages. Providing further support for a direct interaction between pathogens and progenitor cells in the bone marrow, Nagai and colleagues demonstrated that HSPCs, particularly early hematopoietic progenitors, expressed TLRs and that TLR signaling through MyD88 could drive the differentiation of myeloid progenitors [99].

## 5. Perspectives

### 5.1. Development of Pathogen-Specific MDSCs of Limited Function

Our data suggests that these TII cells protect not only against polymicrobial IAI, but also against bloodstream infections (BSIs) with *C. albicans*. Whether these cells provide protection against other BSIs or in other models of polymicrobial sepsis remains to be determined. It is tempting, however, to speculate that *C. dubliniensis*-induced MDSCs may provide pathogen-specific protection via the upregulation of different repertoires of pattern recognition receptors (PRRs), similar to what has been proposed for gram-negative versus gram-positive bacteria [100]. 

**Figure 2:**
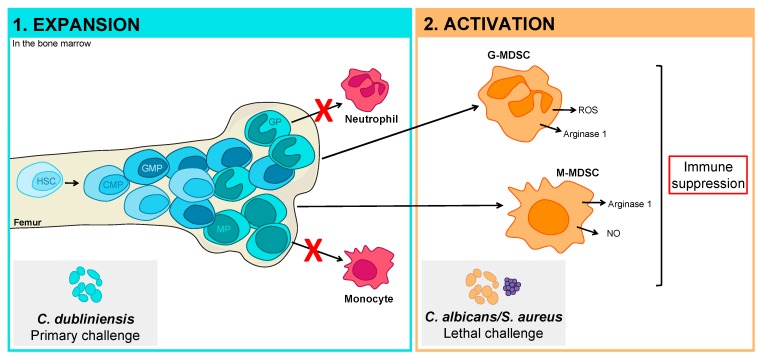
Model of myeloid-derived suppressor cells (MDSC) expansion and activation steps during *C. dubliniensis*-mediated protection against polymicrobial sepsis. We propose that *C. dubliniensis* in the bone marrow during the primary challenge induces the expansion of the immature myeloid population and a block in normal myeloid cell differentiation. This population is then activated by the *C. albicans*/*S. aureus* lethal challenge to produce mature MDSCs that express their characteristic effectors, resulting in the suppression of detrimental immune responses and protection against lethal sepsis. HSC, hematopoietic stem cell; CMP, common myeloid progenitor; GMP, granulocyte-monocyte progenitor; GP, granulocytic precursor; MP, monocytic precursor; G-MDSC, granulocytic MDSC; M-MDSC, monocytic MDSC.

Based on the above work characterizing how pathogens can manipulate the hematopoietic compartment, combined with our understanding of how MDSCs are developed, we hypothesize that MDSC-mediated protection against polymicrobial IAI is initiated by *C. dubliniensis* in the bone marrow (Figure 2). Based on the two-signal model of MDSC development [38], this would represent the MDSC expansion step, in which the immature myeloid population is expanded in the bone marrow and normal differentiation is blocked. We then hypothesize that *C. albicans*/*S. aureus* lethal challenge represents the activation step, in which the immature myeloid cells are converted to fully functioning MDSCs. Whether this occurs in the bone marrow as well remains to be determined. We have demonstrated that *C. albicans* and *S. aureus* can access the bone marrow compartment [85], however, they are more rapidly cleared than other protective *Candida* species. Alternatively, the expanded immature myeloid population may be recruited to sites of inflammation associated with *C. albicans*/*S. aureus* infection, at which time they become activated and suppressive. 

### 5.2. Trained Tolerogenic Immunity

Netea and colleagues introduced the concept of trained innate immunity based on the fact that plants and several invertebrates, which lack adaptive immunity, have mechanisms of protection against secondary infections [24]. Furthermore, several studies had previously demonstrated protection in mammals that was independent of adaptive immunity. In the late 1980s, Bistoni et al. demonstrated that a low virulence, yeast-locked *C. albicans* strain could provide protection against infection with its virulent counterpart [101,102]. They further demonstrated that this protection was independent of T and B cells, with protection instead conferred by “plastic-adherent” mononuclear cells, presumably macrophages. Similarly, van’t Wout et al. showed that the BCG vaccination could provide protection against *C. albicans* that was macrophage-mediated [103]. In more recent years, long-lived, self-renewing natural killer (NK) cells with adaptive immune properties have also been described, which can provide protection against viral challenge [104]. Netea and colleagues have demonstrated that *C. albicans* infection, or *C. albicans* cell wall-derived β-glucan, can protect against reinfection in a T and B cell-independent manner through the training of monocytes [105]. This training has been shown to occur through the epigenetic reprogramming of genes involved in cytokine production and metabolism [105,106,107,108]. A plethora of in vitro studies have demonstrated that *C. albicans* trains and reprograms monocytes to induce a more robust response to secondary exposure, including increased inflammatory cytokine production and phagocytosis, which leads to improved antifungal activity and survival. However, we demonstrated that macrophages were not involved in the *C. dubliniensis*-mediated protection [23]. Furthermore, until our discovery, neither Gr-1^+^ PMNs nor MDSCs had been previously reported to play a role in trained innate immunity.

Endotoxin tolerance (ET) is defined as the reduced capacity of a cell to respond to LPS/endotoxin after an initial exposure to it [109,110]. ET is characterized by a downregulation of inflammatory mediators, including TNFα, IL-1β, and CXCL10, and the upregulation of anti-inflammatory factors, such as IL-10 and TGF-β [109]. It is thought to be a regulatory mechanism for the host to combat overabundant inflammation. Similar to TII, tolerized monocytes undergo a functional reprogramming that is driven by epigenetic modifications, however, unlike TII, ET induces epigenetic modifications that result primarily in gene silencing [107,111,112]. Tolerized monocytes become more anti-inflammatory and have enhanced phagocytosis and antimicrobial activity [111,113,114,115]. Endotoxin tolerance is closely related to the compensatory anti-inflammatory syndrome (CARS) that is observed in sepsis patients [116]. 

**Figure 3:**
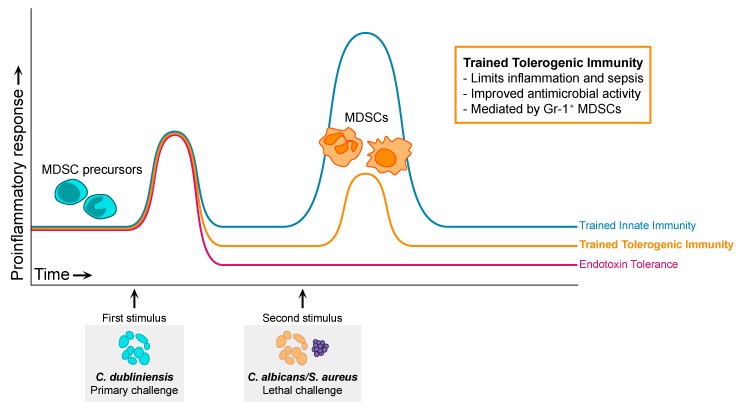
Graphical representation of primary and secondary immune responses associated with the proposed spectrum of trained innate memory, including trained tolerogenic immunity (TTI), trained innate immunity (TII), and endotoxin tolerance (ET). Adapted from [117].

The *C. dubliniensis*/MDSC-mediated protection against polymicrobial sepsis that we have described shares features of both trained innate immunity and endotoxin tolerance. However, several pieces of data suggest that this is a distinct form of trained innate memory. Unlike TII, which has mostly been described for monocytes/macrophages and NK cells, our data suggests that protection is mediated by MDSCs. Furthermore, in contrast to the direct effector function of the trained cells in TII, we hypothesize that protection by MDSCs is mediated by suppression of the pathological sepsis-associated inflammatory response, similar to the anti-inflammatory state induced by immune cell reprograming in ET. But unlike ET, MDSC-mediated immune suppression in our model is beneficial to the host. Therefore, we suggest that there is a spectrum of trained innate memory (Figure 3), from TII, representing enhanced, beneficial, secondary inflammatory responses, to ET, demonstrating a detrimental lack of secondary response. Falling in the middle of this spectrum is the MDSC-mediated secondary response in our model that leads to beneficial suppression, for which we propose the term “trained tolerogenic immunity” (TTI). Whether the mechanism of protection conferred by trained MDSCs includes robust antifungal defenses, similar to trained monocytes, or is limited to direct suppression of the sepsis proinflammatory response, similar to ET, remains to be determined and will be discussed in the following section. 

### 5.3. Mechanisms of MDSC Protection in Sepsis

There are several questions that remain to be answered with respect to *C. dubliniensis*- and MDSC-mediated TTI. While we observed that *C. albicans* was able to access the bone marrow, the lack of protection suggests that *C. albicans* is unable to induce the expansion of MDSCs. This may be because *C. albicans*, as a more virulent species, is too damaging to the HSPCs in the bone marrow, inhibiting their ability to expand and develop into MDSCs. The fact that several virulent *Candida* species are unable to provide protection in our model suggests a damage hypothesis, in which low damage or low virulence is associated with protection. Another aspect to consider is timing. Several studies have shown that MDSCs require an extended period of time to develop and become fully mature [82,83]. It is also possible that the damage caused by and/or the robust inflammatory response to *C. albicans* and other virulent strains results in animal death before MDSCs can develop. In this case, *C. albicans* can effectively initiate MDSC expansion, but the animals succumb before they can be activated. By contrast, *C. dubliniensis* and other low virulence species do not induce robust inflammation or rapid animal death, allowing time for MDSCs to expand and become activated. 

Another important open question is how protection is mediated to enhance survival. One possibility is that the MDSCs are directly killing *C. albicans* and/or *S. aureus* in addition to mediating canonical suppression of the septic proinflammatory response. Both TII and ET point towards this possibility, as trained and tolerogenic monocytes have been shown to be more antimicrobial with increased levels of phagocytosis. Rieber et al. also demonstrated that fungal-induced MDSCs that were protective against candidiasis were more antifungal, however, they concluded that this function was a relatively minor contribution to overall protection [77]. Another possibility is that *C. dubliniensis*-trained MDSCs suppress the septic response and also differentiate into other innate cells, which ultimately kill *C. albicans*/*S. aureus*. Several studies have demonstrated that MDSCs may retain their ability to differentiate into mature innate cells [37,118]. Furthermore, MDSCs in tumor models have been shown to be able to differentiate into tumor-associated macrophages (TAMs) [119]. A third possibility is that primary protection is driven by the MDSC-mediated suppression of the septic response, while antimicrobial activity is mediated by a classical innate response (PMNs, macrophages). In this regard, it stands to reason that if the lethal septic inflammatory response is suppressed, the classical innate cells would have time to function normally to reduce the source of infection. 

## 6. Conclusions

Polymicrobial infections are increasingly common and difficult to combat. In particular, the contributions of fungi are often overlooked, however, their impact on these infections is significant. We have identified a novel form of TII induced by *C. dubliniensis* that can provide protection against fungal-bacterial IAIs. This protection is mediated by MDSCs, which have been identified in both sepsis and fungal infections, but their role in TII has not been described previously. We propose that MDSC-mediated protection against polymicrobial sepsis falls along the spectrum of trained innate memory, with protective responses associated with the suppression of pathological inflammation representing trained tolerogenic immunity (TTI). Future work is aimed at understanding how MDSCs develop in response to *C. dubliniensis* and how they provide protection in models of polymicrobial IAI.
